# The Effects of a Ketogenic Medium-Chain Triglyceride Diet on the Feces in Dogs With Idiopathic Epilepsy

**DOI:** 10.3389/fvets.2020.541547

**Published:** 2020-12-22

**Authors:** Rachel Pilla, Tsz Hong Law, Yuanlong Pan, Brian M. Zanghi, Qinghong Li, Elizabeth J. Want, Jonathan A. Lidbury, Joerg M. Steiner, Jan S. Suchodolski, Holger Andreas Volk

**Affiliations:** ^1^Gastrointestinal Laboratory, Department of Small Animal Clinical Sciences, Texas A&M University, College Station, TX, United States; ^2^Department of Clinical Science and Services, Royal Veterinary College, Hatfield, United Kingdom; ^3^Nestlé Purina Research, One Checkerboard Square, St. Louis, MO, United States; ^4^Division of Integrative Systems Medicine and Digestive Disease, Imperial College, London, United Kingdom; ^5^Small Animal Clinic, University of Veterinary Medicine, Hanover, Germany

**Keywords:** epilepsy, dogs, microbiome, ketogenic diet, medium-chain triglyceride

## Abstract

Consumption of diets containing medium chain triglycerides have been shown to confer neuroprotective and behavior modulating effects. We aimed to identify metabolic and microbiome perturbations in feces that are associated with consumption of a medium chain triglyceride ketogenic diet (MCT-KD) in dogs with idiopathic epilepsy. We used 16S rRNA gene sequencing to generate microbiome profiles and ultra-performance liquid chromatography-mass spectrometry (UPLC-MS) to generate lipidomic profiles of canine feces. We made comparisons between the MCT-KD, standardized placebo diet and baseline pre-trial diet phases. Consumption of the MCT-KD resulted in a significant increase in the species richness (α-diversity) of bacterial communities found in the feces when compared to the baseline diet. However, phylogenetical diversity between samples (beta-diversity) was not affected by diet. An unnamed *Bacteroidaceae* species within genus *5-7N15* was identified by LEfSe as a potential biomarker associated with consumption of the MCT-KD, showing an increased abundance (*p* = 0.005, *q* = 0.230) during consumption of MCT-KD. In addition, unclassified members of families *Erysipelotrichaceae* (*p* = 0.013, *q* = 0.335) and *Fusobacteriaceae* (*p* = 0.022, *q* = 0.358) were significantly increased during MCT-KD consumption compared to baseline. *Blautia* sp. and *Megamonas* sp. instead were decreased during consumption of either placebo or MCT-KD (*p* = 0.045, *q* = 0.449, and *p* = 0.039, *q* = 0.449, respectively). *Bacteroidaceae*, including genus *5-7N15*, have previously been associated with non-aggressive behavior in dogs. In addition, *5-7N15* is correlated in humans with *Akkermansia*, a genus known to be involved in the neuroprotective effect of ketogenic diets in mice models of seizures. Five metabolite features, tentatively identified as long chain triglycerides, were significantly higher after consumption of the placebo diet, but no unique features were identified after consumption of the MCT-KD. The data presented in this study highlight significant changes shown in both the fecal microbiome and lipidome as a result of consumption of the MCT-KD. Elucidating the global canine gut response to MCT consumption will improve our understanding of the potential mechanisms which confer anti-seizure and behavior modulating effects. Further studies should aim to characterize the gut microbiome of both dogs with epilepsy and healthy controls.

## Introduction

Epilepsy is a common chronic neurological disorder in dogs with an estimated prevalence of 0.6–0.75% in first opinion practice (1, 2). Epilepsy in dogs has been associated with increased risk of premature and unexpected death, injuries, neurobehavioral, and cognitive dysfunction and reduced quality of life (3–5). Despite ongoing research in understanding the biological manifestations of seizures and epilepsy, the cellular pathologies remain elusive. Approaches toward therapy for dogs with epilepsy has been limited toward the control of seizures, most commonly chronic administration of anti-seizure drugs (ASDs), rather than prevention of epileptogenesis. Despite appropriate ASD treatment, often incorporating multiple drugs, approximately one third of dogs with idiopathic epilepsy (IE) continue to experience seizures that are difficult to control. Furthermore, ASD-related side effects such as ataxia, sedation, polyphagia, polyuria and polydipsia in dogs contribute to reduction in quality of life (6). This emphasizes the importance of new treatment strategies to improve the welfare of dogs with epilepsy.

The use of dietary manipulations, such as ketogenic diets (KD) to prevent seizures in humans with epilepsy, was first introduced dating back to the 1920s, whereby the primary aim was to emulate biochemical changes associated with fasting (7, 8). Since then different dietary methods have consistently reported anticonvulsant properties to varying degrees in humans with epilepsy (9–12). It is thought that canine epilepsy is not only naturally occurring but also similar in pathology, heterogeneity, etiology, and clinical manifestations to its human counterpart and thus acts as a good translational model (13–15). In 2015, Law et al. reported the anti-seizure properties of a medium chain triglyceride (MCT) ketogenic diet (MCT-KD) in a 6 month, placebo controlled crossover diet trial in dogs with IE (16). The MCT-KD contained 5.5% medium chain triglycerides (MCT) and while containing 50% carbohydrate content, still resulted in a significant rise in blood β-hydroxybutyrate (BHB) concentrations (16). The MCTs included in the diet are able to induce ketosis more readily than long chain triglycerides (LCT) found in other dietary fats, therefore allowing for more carbohydrates and proteins within the diet, which in effect also increases diet palatability (17, 18). There has been a consistent flow of scientific literature corroborating anti-seizure effects possessed by different ketogenic dietary methods, and as such, a progressive development of different hypotheses explaining the therapeutic mechanism of action (19, 20). Unfortunately, to date, the exact mechanisms of action remain unknown.

There has been increasing attention and evidence highlighting the importance of the gut microbiome in epilepsy (21, 22). In one human patient who had been experiencing seizures for 17 years, a fecal microbiota transplant (FMT), resulted in complete seizure freedom despite discontinuing ASD treatment during the 20-month follow-up period (23). In another study, six patients with drug resistant epilepsy achieved significant seizure frequency reduction, five experiencing complete seizure freedom, during antibiotic treatment (24). Interestingly, the patients were not all treated with the same antibiotics, hence reducing the likelihood that anti-seizure effects were drug interaction related but instead as an outcome of microbiota depletion. Peng et al., also showed that the microbiome composition correlated with drug responsiveness, where drug-resistant patients expressed increased abundance of rare flora whereas drug-sensitive patients expressed a microbiome composition similar to healthy controls (25).

The composition of the gut microbiome is influenced by environmental factors more so than by host genetics, where diet represents the predominate environmental factor influencing the makeup of the intestinal microbiome community (26). The influence of the gut microbiome has not only been highlighted in patients with epilepsy and seizures, but also in the anti-seizure efficacy of KDs (27). Olson et al. found that a 6:1 fat:protein KD selectively enriched *Akkermansia* and *Parabacteroides* in mice, and the enrichment of those two bacteria genus alone was enough to confer seizure protection (27). This protective effect was transferable to other mice fed a normal diet by colonizing their gut with fecal microbiota from mice fed the KD.

Considering the potential that mechanisms by which KDs confer anti-seizure effects in dogs via influences to the gut microbiome, fecal samples obtained from a previously published diet trial by Law et al. have been used in this study (16). The 6-month, placebo-controlled cross-over dietary trial was designed to compare the MCT-KD with a standardized placebo diet in chronically ASD-treated dogs with idiopathic epilepsy (IE). Seizure frequency was shown to be significantly reduced when dogs were fed the MCT-KD when compared to the placebo diet (16). Interestingly, the MCT-KD was also previously shown to confer significant behavior modifications in the dogs with IE (28). The aim of this study was to evaluate changes in the gut microbiome and lipidome of dogs fed a MCT-KD and make further comparisons between paired pre-trial and placebo-fed fecal profiles.

## Materials and Methods

This study was conducted in accordance with the guidelines laid down in the VICH (International Cooperation on Harmonization of Technical Requirements for Registration of Veterinary Medical Products) GL9 GCP (Good Clinical Practices) and the EMEA (European Agency for the Evaluation of Medical Products). The studies involving animals were reviewed and approved by the Royal Veterinary College Ethics and Welfare Group (URN 2011 1132). The owners of the dogs gave consent for their animals to be used in this study. The 6-month, randomized, double blinded, placebo controlled, cross-over, MCT-KD trial study for dogs with idiopathic epilepsy has previously been reported (16). The study population consisted of 21 dogs with a mean of 4.59 (*SD* 1.73) years of age and weighed a mean of 29.79 (*SD* 14.73) kg at the start of the trial (Supplementary Table 1). For the current study, 10 dogs were excluded due to confounding factors that could impact microbiome composition. These factors, unrelated to participation to the diet trial, included systemic antibiotic usage (4 dogs), gastrointestinal symptoms requiring treatment (1 dog), or both (5 dogs) at any time during the diet trial [(16); Supplementary Table 1].

### Diets

Dogs were fed either the MCT-KD or the placebo diet for 3 months (day 1 to day 90 ± 2 days) followed directly by a subsequent respective switch of diet for a further 3 months (day 90 to day 180 ± 2 days). The normal diet in this study refers to the diet normally fed to each of the dogs by owners at home. The experimental placebo and MCT-KD formulas were dry extruded kibble (Nestle Purina PetCare, St. Louis, Missouri, USA) and compositions reported in Table 1. The placebo formula contained zero MCTs and lard was used as fat substitute, whereas the test formula contained 5.5% MCT oil. MCT/lard content was about 10% of total formula calories (based on lard as 8.5 kcal/g and MCT as 6.8 kcal/g).

**Table 1:** Composition of diets.

	**Placebo**	**MCT**
**Nutrient composition (% as fed)**		
Crude protein	27.3	29.7
Crude fat	13.4	14.2
Carbohydrate	40.4	39.7
Crude fiber	4.6	2.9
MCTs	0	5.5^*^
Moisture	8.8	8.1
Ash	5.5	5.4
**Energy content**		
Calculated ME (kcal/kg)^**^	3,510	3,630

** *Calculated based on the predictive equation for metabolizable energy in dog foods (29)*.

* *MCTs were included as part of the crude fat*.

### Ketone Body Measurement

Blood samples were collected from the dogs over the duration of the study corresponding to MCT-KD and placebo diet samples on the days of visit [end of period 1—day 90 (*SD* 2) d and end of period 2—day 180 (*SD* 2) d]. Blood samples were collected 2 h after consumption of the respective diets and routine concomitant AEDs. Blood samples were collected and stored using clotting activator dipotassium EDTA-containing (with serum-separation gel) polypropylene blood-collection tubes and plain polypropylene blood-collection tubes (International Scientific Supplies Ltd.). Blood samples were allowed to clot and serum was stored at −80°C. Serum samples were analyzed for β-hydroxybutyrate (BHB) concentrations using an enzymatic end point (colorimetric) reaction assay as directed by following the kit manufacturer's protocol (C444-0A; Catachem Inc.) and using an Olympus AU 640e (Beckman Coulter) chemistry analyser for measurements.

### Sample Collection and Preparation

Naturally voided fecal samples were collected from the dogs over the duration of the study at visit one (day 0), at visit two on day 90 (±2 days), and at visit three on day 180 (±2 days), corresponding to baseline diet phase, placebo diet phase or MCT-KD phase samples. Fecal samples were frozen immediately after collection and transported on dry ice. Fecal samples were thawed on ice prior to sample preparation for 16s rRNA sequencing and metabolic profiling.

#### 16S rRNA Sequencing

For 16S rRNA sequencing, DNA extraction was achieved using a Biostatic Bacteremia DNA Isolation Kit (MoBio Laboratories, USA) as per manufacturer's protocols. Unique sequence barcodes were used to facilitate identification of samples. Fusion Primers (forward and reverse primers), purchased from Integrated DNA technologies, were used to facilitate amplification of extracted DNA by PCR. Subsequent removal of primer regions and unwanted small DNA fragments was performed using a Purelink® Pro 96 Purification Kit as per manufacturer's protocols (Life technologies Corporation). Pooled DNA sequences were normalized, further amplified via emulsion PCR (emPCR; Roche Diagnostics) as per manufacturer's protocols. The number of emPCR beads were normalized ready for sequencing (Supplementary Table 2).

#### LC-MS Metabolic Profiling

For UPLC-MS profiling, fecal samples were homogenized using a Precellys bead beater with pre-chilled methanol/ water (1:1) and zirconia silica beads (BioSpec Products Inc., USA). Two-thirds of supernatant was removed for other experimental methods while the remaining was combined with pre-chilled dichloromethane/methanol (3:1) and homogenized further using the Precellys bead beater. Supernatants were dried using a centrifugal evaporator and re-suspended in methanol/water (1:1) for UPLC-MS analysis [(30); Supplementary Table 2]. Pooled quality control (QC) samples were made using 50 μl of each study sample (30).

### Data Acquisition and Extraction

#### 16S rRNA Sequencing

DNA sequencing was performed using a 454 GS-FLX+ Genome Sequencer (Roche Diagnostics) as per manufacturer's protocols. DNA sequence data were generated and exported in the FASTA file format and subsequently processed and analyzed using Quantitative Insights Into Microbial Ecology [QIIME) v 1.9 (31)]. Raw DNA sequences were uploaded to NCBI Sequence Read Archive under the accession number SRP162687. The sequence data were de-multiplexed and quality filtered using the default settings for QIIME. Chimera detection and filtering was performed using USEARCH against the 97% clustered representative sequences from the Greengenes v 13.8 database (32). The remaining sequences were clustered into Operational Taxonomic Units (OTUs) using an open reference approach in QIIME. Prior to downstream analysis, sequences assigned as chloroplast, mitochondria, and low abundance OTUs, containing <0.01% of the total reads in the dataset were removed. All samples were rarefied to 2,940 sequences per sample to account for unequal sequencing depth. The rarefaction depth was chosen based on the lowest read depth of samples. Alpha diversity was measured with the Chao1 (richness), Shannon diversity, and observed species metrics. Beta diversity was evaluated with the phylogeny based UniFrac distance metric and visualized using Principal Coordinate Analysis (PCoA) plots (33). Linear discriminant analysis effect size (LEfSe) was used to elucidate bacterial taxa that were associated with each diet trial (34). LEfSe was calculated using Calypso, a web-based software package that allows mining and visualizing of microbiome-host interactions (35).

#### UPLC-MS Lipid Profiling

UPLC-MS analysis was performed using a Charged Surface Hybrid (CSH) column on an Acquity UPLC system (Waters Corporation, USA). Mass spectrometry was carried out on a Q-TOF Premier mass spectrometer (Waters MS technologies, UK). Data extraction was performed by peak picking and grouping using the XCMS package in R programming language [open-source software (R, v3.3.2)]. The metabolite features list which passed all quality control filtering protocols (30), were normalized by median fold change using an in house normalization script executed in the R programming language [open source software, (R, v3.3.2)]. Further information on the metabolic profiling parameters can be found in Supplementary Table 3.

### Statistical Analysis

#### 16S rRNA Sequencing

Statistical analysis of microbiome data was performed using GraphPad Prism v7.0. Statistics using 2-way ANOVA was performed for alpha-diversity comparisons, and for each individual bacterial taxon detected. *P*-values were corrected for false discoveries using Benjamini and Hochberg's False Discovery Rate. An adjusted *p*-value (*q*-value) <0.05 was considered statistically significant. Tukey's multiple comparison test was used to determine the pairwise comparisons that were significantly altered by diet. Multivariate statistics were performed on beta-diversity data using Analysis of Similarity test (ANOSIM), which was performed with PRIMER 6 software package (PRIMER-E Ltd., Luton, UK), to analyze significant differences in microbial communities between the control diet and the two food trials.

#### UPLC-MS Lipid Profiling

Statistical analysis of canine fecal data included an overview examination of data using MVDA (multivariate data analysis) techniques, performed using SIMCA-P+ (v14.0.01359, Umetrics) software, followed by subsequent univariate data statistical analysis (UVDA), performed using R programming language [(R, v3.3.2) open-source software]. All metabolite features, extracted, and included in further data analysis, were subject to paired student *t*-test analysis followed by false discovery rate (FDR) multiple *t*-test, *p*-value correction (36). Significant metabolites (*p* < 0.05) were carried forward for further structural identification (ID) by UPLC-tandem mass spectrometry (MS/MS) analysis. Relative metabolite abundance fold change was calculated based on overall average of individual fold changes for each paired sample and was relative to the lower abundance diet phase group. UPLC-MS/MS data were collected with collision energy at 10 and 50 V in RP and at 10 and 40 V in lipid profiling. Metabolite identification was facilitated by mass spectra fragmentation patterns and matching mass-to-charge ratio (m/z) to metabolites found in online databases including; LIPID MAPS (http://www.lipidmaps.org); HMDB (http://www.hmdb.ca/); METLIN (https://metlin.scripps.edu/index.php); and other published literature (37). Subsequent UPLC-MS/MS ID verification required a mass match (m/z) of ±0.01 m/z and a retention time match of 0.5 min to the original detected metabolite feature to be considered significant.

#### Clinical Outcome

Comparisons between the MCT-KD and placebo-standardized diet groups were made using two-sided match-paired Student's *t*-tests for parametric data and two-sided Wilcoxon matched-pairs signed rank test for non-parametric data. Non-parametric data are presented as median (25th−75th percentile), and parametric data are presented as mean values and standard deviations.

## Results

### Clinical Outcome

**Figure 1:**
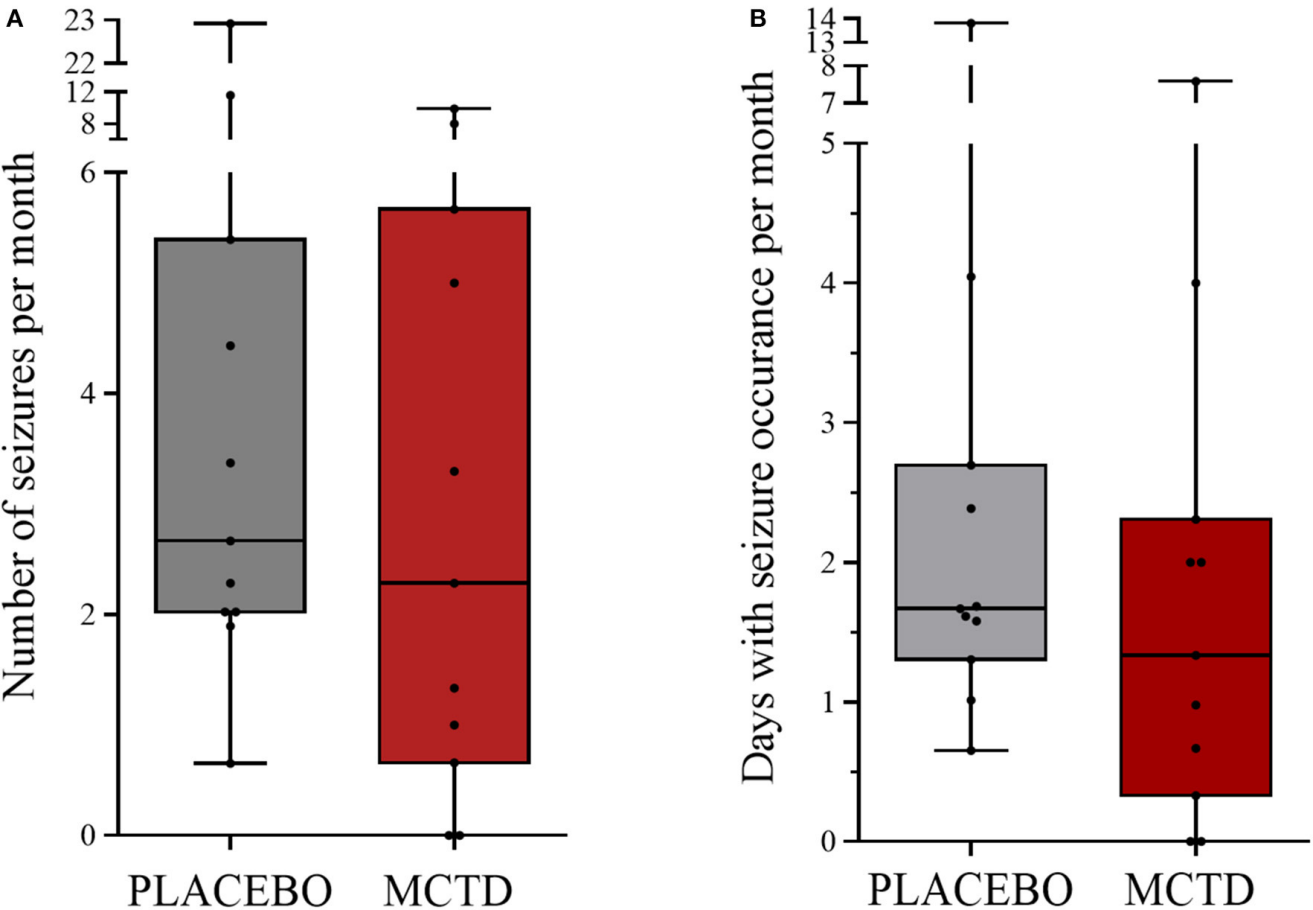
Effect of the medium-chain TAG ketogenic diet (MCT-KD) on **(A)** seizure frequencies per month (*P* = 0.148) and **(B)** seizure days per month (*P* = 0.054) compared with the placebo-standardized diet (*n* = 11). Data are shown as box-and-whisker plots (central lines of the box represent the median, lower and upper limits of the box represent the 25th and 75th percentiles and whiskers represent the minimum and maximum). Two-sided Wilcoxon's matched-pairs rank tests were used to compare placebo and MCTD groups.

**Figure 2:**
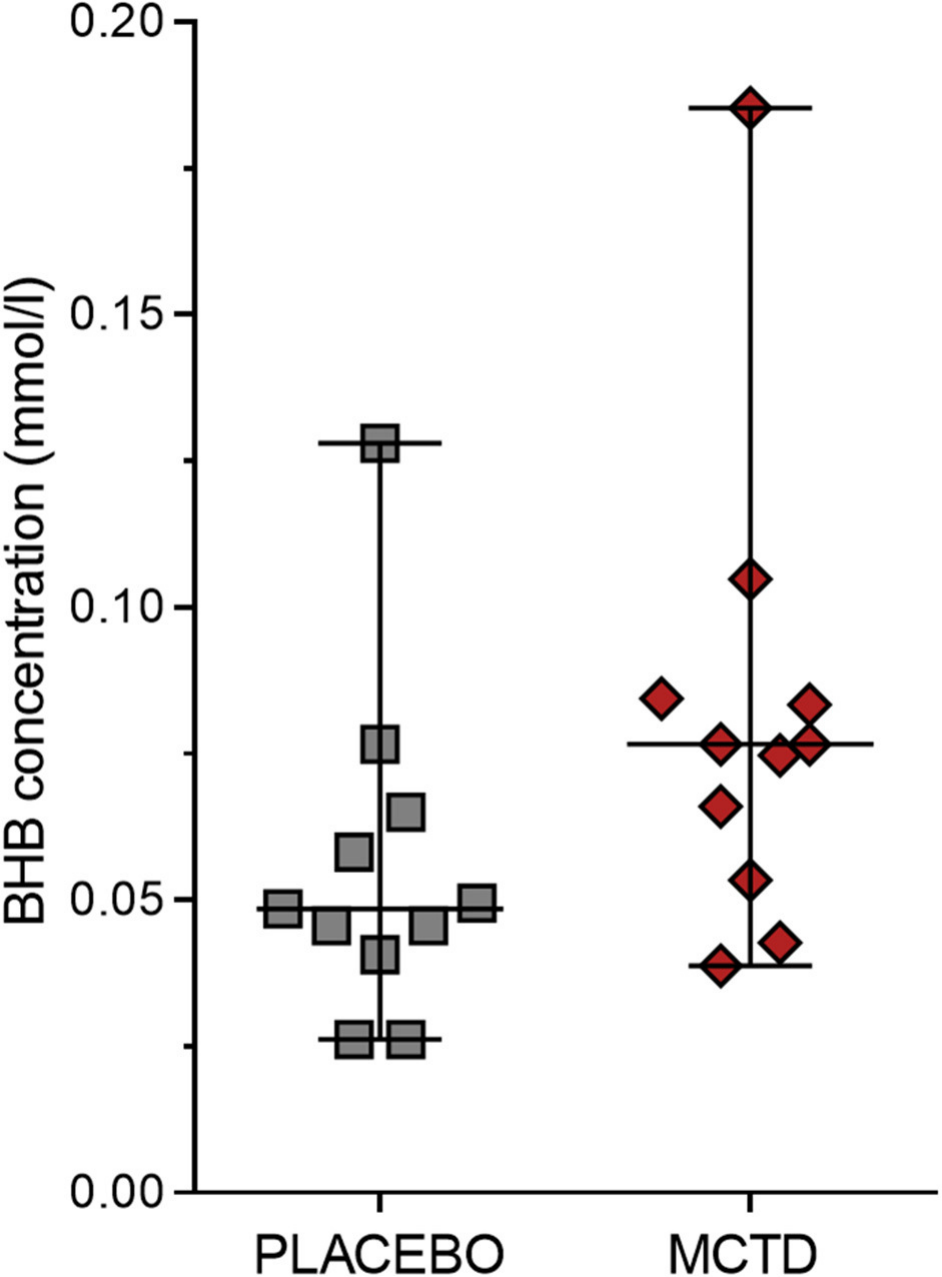
Effects of the medium chain triglyceride diet (MCT-KD) on concentrations of β-hydroxybutyrate (BHB). BHB concentrations were measured after dogs (n = 11) were fed the MCT-KD for a period 90 ± 2 days and the placebo diet for a period 90 ± 2 days. Figure shows a respective increase in BHB levels when dogs were on the MCT-KD in comparison to the placebo diet (p = 0.0498). Data are shown as scatter plot (central line represent mean with standard deviations). Two-sided matched paired students t-test was used to compare the placebo and MCT-KD group.

This study population included 7 males, of which 4 were neutered and 3 were intact, and 4 females, of which 3 were neutered and 1 was intact. The dogs had a mean age of 4.34 (*SD* 2.25) years and a mean weight of 27.52 (*SD* 10.51) kg at the start of the trial (Supplementary Table 1). There was a general reduction in seizure frequency when dogs were on the MCT-KD (2.28/month, 0.66–5.67/month) when compared to the placebo diet (2.67/month, 2.02–5.39/month, *p* = 0.148), Figure 1. Of the eleven dogs included in this study, eight dogs had a reduction in seizure frequency per month during the MCT-KD when compared to the placebo diet; of which 2 dogs achieved seizure freedom, 4 dogs had ≥50% reduction in seizure frequency, 2 dogs had an overall <50% reduction in seizure frequency. Three dogs had no response, with an increase in seizure frequency. There was also a general reduction in the number of days with seizure occurrence per month when dogs were on the MCT-KD (1.33/month, 0.33–2.31/month) when compared to the placebo diet (1.67/month, 1.30–2.70/month, *p* = 0.054), Figure 1. Of the eleven dogs included in this study, 9 had a reduction in number of days with seizure occurrence; 2 dogs achieved seizure freedom, 2 dogs had >50% reduction in seizure days, 5 dogs had <50% reduction in seizure days. Two dogs showed no response, with a general increase in number of seizure days (Supplementary Table 4). Consumption of the MCT-KD resulted in significant elevation of blood β-hydroxybutyrate concentrations in comparison with placebo diet [0.055 mmol/l (*SD* 0.028) vs. 0.081 mmol/l (*SD* 0.040), *p* = 0.050], Figure 2.

### 16S rRNA Sequencing

The sequencing yielded 195,358 quality sequences for all analyzed samples (*n* = 32, median 6,178, range 2,945–7,306) after removing chimeras, and singletons. The samples were rarefied to an equal sequencing depth of 2,940 reads per sample.

### Effect of Diets on Fecal Bacterial Diversity

**Figure 3:**
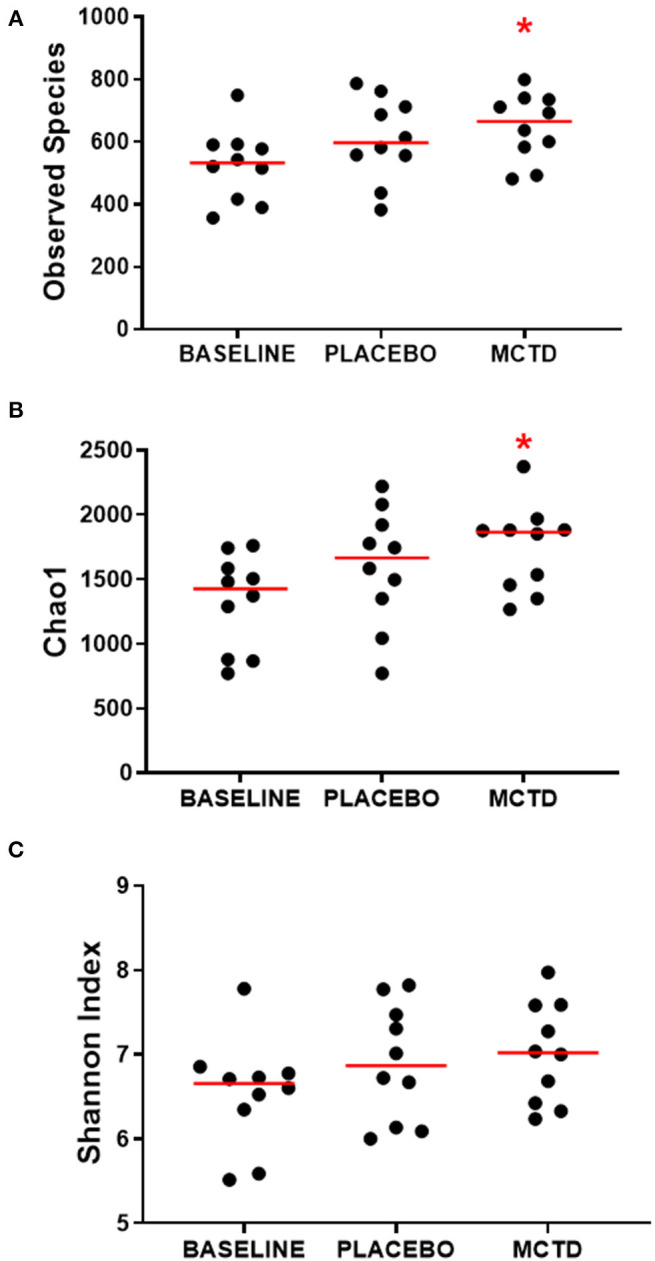
Alpha diversity indices Observed species **(A)**, Chao1 **(B)**, and Shannon **(C)**, measured before (normal) and after diet trial with placebo or MCT-KD diets. Red bars indicate the median, red asterisk indicates significance (*p* < 0.05) compared to baseline.

There was a significant increase in alpha diversity, i.e., within sample diversity, during the MTC-KD period when compared to the day-0 sample, which is representative of all dogs on their respective pre-trial diet (baseline diet). Observed species, defined by the number of OTUs observed in each sample, was significantly increased during the MCT-KD period when compared to baseline diet (MCT-KD: median 664.5, range 481–799; baseline diet: median 532, range 356–749, *p* = 0.025, Figure 3A). There was no statistical significance between the MCT-KD and placebo diet (*p* = 0.622), or between placebo and baseline (*p* = 0.170).

Similarly, the Chao1 non-parametric estimator of species richness also showed a significant increase in alpha diversity during the MCT-KD period when compared to the pre-trial baseline diet (*p* = 0.025, Figure 3B). There was no statistical significance between the MCT-KD and placebo diet (*p* = 0.588), or between placebo and baseline (*p* = 0.170). Although there were no significant changes in the Shannon diversity index, which accounts for both species richness and evenness, a similar trend could be seen (Figure 3C).

**Figure 4:**
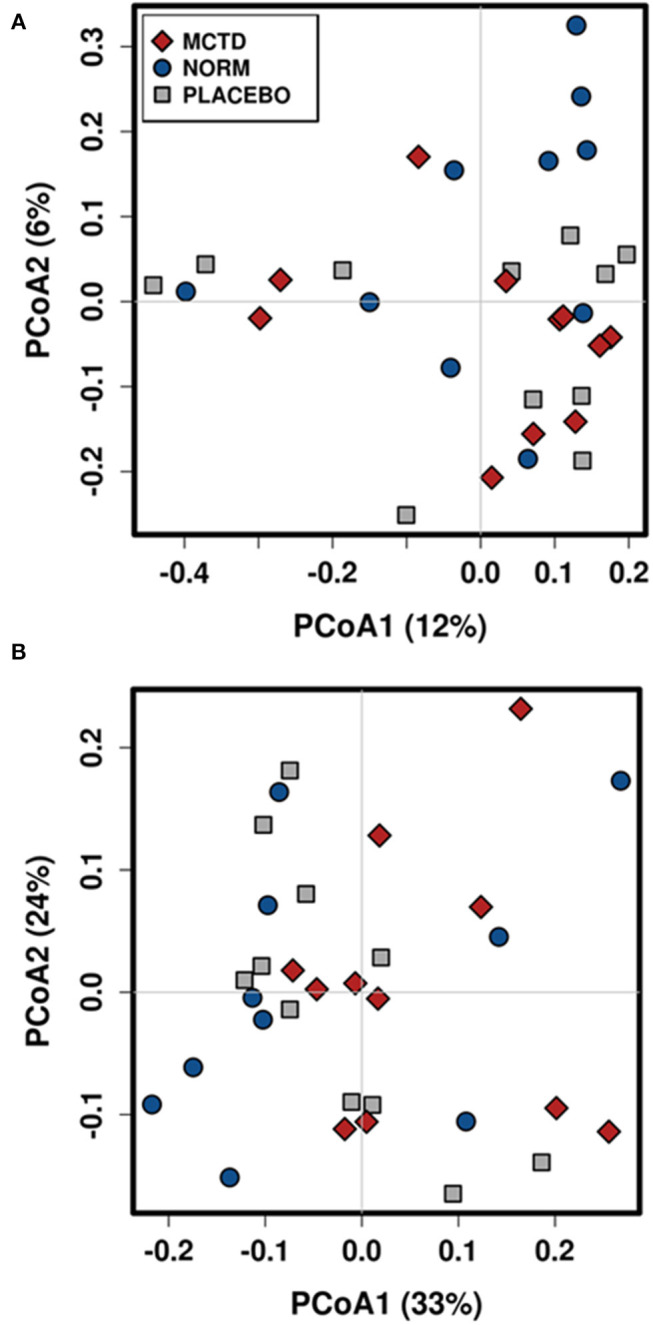
Principal Coordinates Analysis (PCoA) of unweighted **(A)** and weighted **(B)** UniFrac distances. Blue circles indicate baseline samples, gray squares indicate samples collected after placebo diet consumption, and red diamonds indicate samples collected after MCT-KD diet consumption.

Diet did no significantly influence the β-diversity (between sample diversity) in bacterial communities which is represented by a lack of clustering in PCoA plots (Figure 4). There were no significant differences between the baseline diet, MCT-KD and placebo diet when considering phylogenetical diversity alone (unweighted UniFrac *p* = 0.313, *R* = 0.015; Figure 4A), or in combination with taxa abundance (weighted UniFrac *p* = 0.106, *R* = 0.048; Figure 4B).

### Linear Discriminant Analysis Effect Size (LEfSe)

Using the LEfSe biomarker discovery algorithm, a few bacterial taxa were shown to be associated with diet (Table 2). Fecal samples from dogs during the MCT-KD were associated with a higher abundance of *Bacteroidaceae* (family), *Bacteroides* (genus), an unclassified *Bacteroides* species, and another unnamed *Bacteroidaceae* species within genus *X57N15*. Fecal samples from dogs during the placebo diet, instead, were associated with a higher abundance of an unclassified genus within the family *Erysipelotrichaceae* and an unclassified *Erysipelotrichaceae* species.

**Table 2:** Linear discriminant analysis of bacterial taxa and their associations with disease.

**Bacterial taxa**	**Diet**	**LDA**
Family *Bacteroidaceae*	MCT-KD	4.85
Genus *Bacteroides*	MCT-KD	4.58
Unclassified species within genus *Bacteroides*	MCT-KD	4.56
Unclassified species within genus *57N15*	MCT-KD	4.27
Unclassified genus within family *Erysipelotrichaceae*	PLACEBO	4.27
Unclassified species within family *Erysipelotrichaceae*	PLACEBO	4.28

*Only a LDA score of >3.5 is shown*.

### Bacterial Taxa Affected by Diet

**Figure 5:**
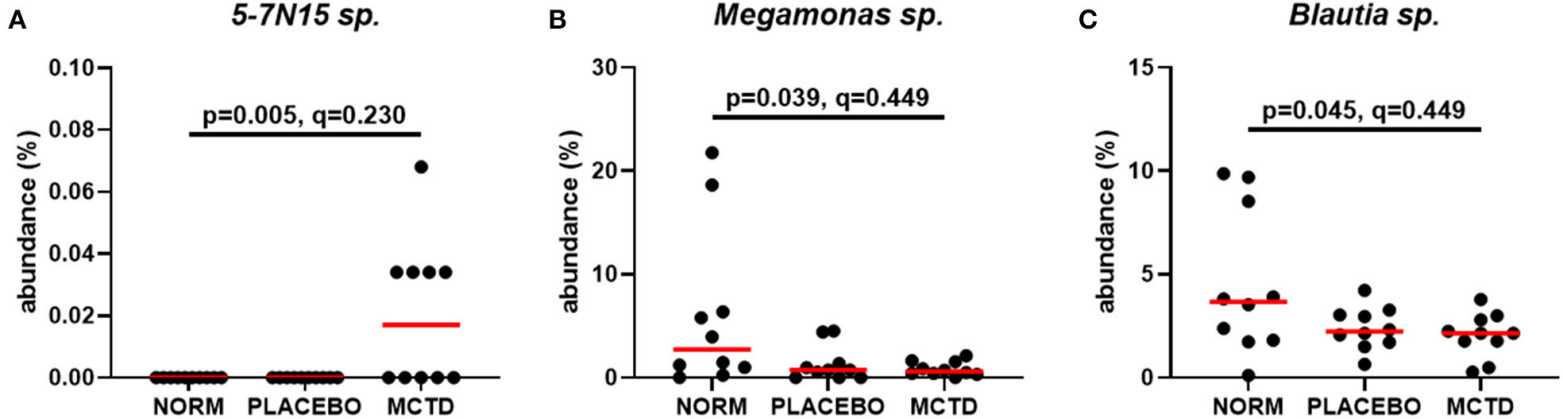
Relative abundance of the most relevant species affected by diet: *5-7N15* sp. **(A)** was increased by MCTD, while *Blautia* sp. **(B)** and *Megamonas* sp. **(C)** showed a trend toward decrease by both the placebo and MCTD diets. Overall *p*- and *q*-values are indicated in the graphs, red bars indicate median.

Statistics revealed bacterial taxa within three phyla that were significantly impacted by the diets, however, significance was lost when *p*-values were adjusted for multiple comparisons (Table 3). Within Bacteroidetes, *5-7N15* sp. was significantly higher in abundance during the MCT-KD (*p* = 0.005, *q* = 0.230, Figure 5A). Within phylum Firmicutes, *Megamonas* sp. (family *Veillonellaceae*) showed a trend toward reduction during both MCT-KD and placebo diet when compared to baseline diet (*p* = 0.039, *q* = 0.449, Figure 5B). A species from genus *Blautia* showed a similar trend (*p* = 0.045, *q* = 0.449, Figure 5C). An unclassified genus within the *Erysipelotrichaceae* family, instead, increased during the placebo diet, and showed a trend toward increasing during the MCT-KD (*p* = 0.013, *q* = 0.275). Within Fusobacteria, one unclassified genus from family *Fusobacteriaceae* was also increased on MCT-KD compared with the baseline diet (*p* = 0.022, *q* = 0.294). Median, range and statistics for all other relevant taxa are included as supplementary materials (Supplementary Table 5).

**Table 3:** Bacterial taxa found to be significantly altered before correction for false discoveries.

**Bacterial taxa**	**Median (%)**			**Range (min–max%)**			**2way ANOVA *p*-value**	**Adjusted *p*-value**
	**Control**	**Placebo**	**MCTD**	**Control**	**Placebo**	**MCTD**		
**Family**								
Veillonellaceae	3.7^a^	0.9^a^	0.9^a^	0–21.9	0–5.9	0.1–3.3	0.039	0.627
**Genus**								
*5-7N15*	0^a^	0^a^	0^b^	0–0	0–0	0–0.1	0.005	0.189
*Megamonas*	2.7^a^	0.7^a^	0.5^a^	0–21.8	0–4.5	0–2.1	0.039	0.400
unidentified Erysipelotrichaceae genus	0.3^a^	2.9^b^	2^a,b^	0–3.3	0.5–9.8	0–9.1	0.013	0.275
unidentified Fusobacteriaceae genus	0^a^	0.1^a,b^	0.1^b^	0–0.2	0–0.2	0–0.3	0.022	0.294
**Species**								
*5-7N15* sp.	0^a^	0^a^	0^b^	0–0	0–0	0–0.1	0.005	0.230
*Blautia* sp.	3.7^a^	2.1^a^	2.1^a^	0.1–9.9	0.6–4.2	0.3–5	0.045	0.449
*Megamonas* sp.	2.7^a^	0.7^a^	0.5^a^	0–21.8	0–4.5	0–2.1	0.039	0.449
unidentified *Erysipelotrichaceae* sp.	0.3^a^	2.9^b^	2^a,b^	0–3.3	0.5–9.8	0–9.1	0.013	0.335
unidentified *Fusobacteriaceae* sp.	0^a^	0.1^a,b^	0.1^b^	0–0.2	0–0.2	0–0.3	0.022	0.358

*Range, median, p-values, and q-values for each bacterial taxa are shown*.

a,b *Means not sharing a common superscript differ (P < 0.05, Tukey's multiple comparisons test)*.

### LC-MS Metabolic Profiling

**Figure 6:**
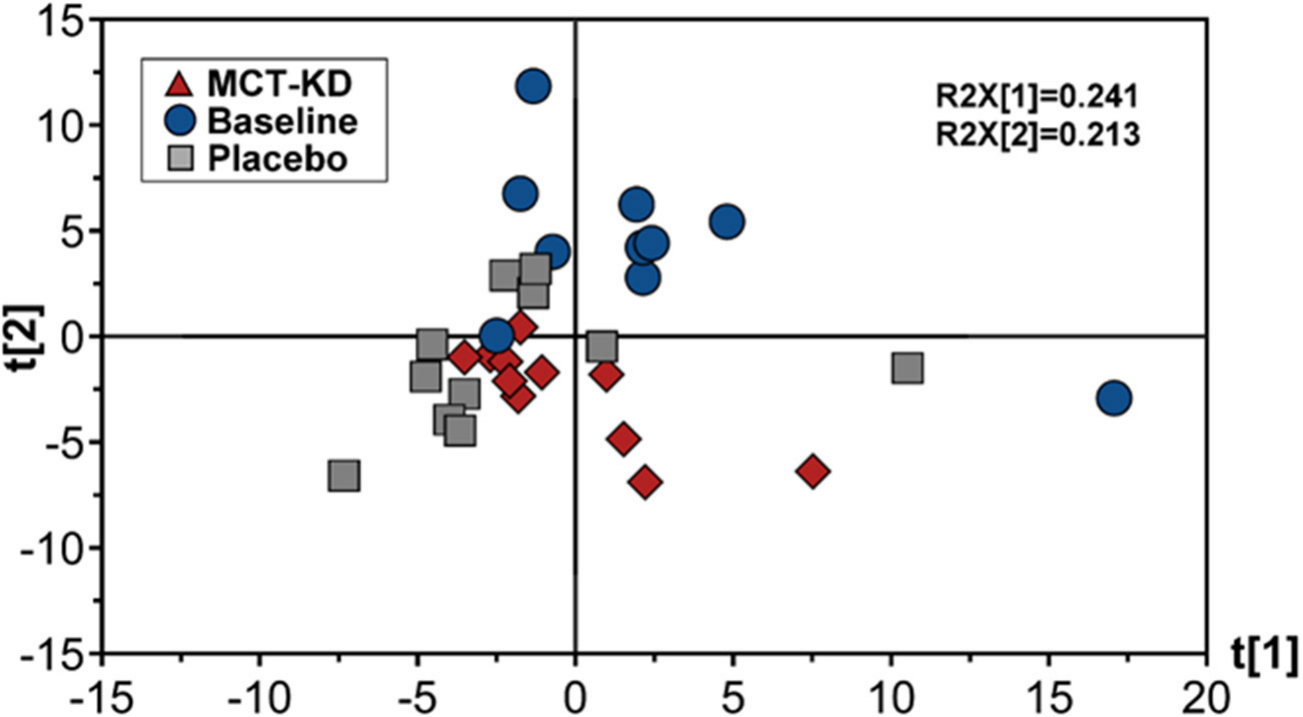
Principal components analysis (PCA) scores plot showing fecal data acquired by lipid profiling UPLC-MS. Scores represent samples belonging to MCT-KD, Placebo and pre-trial baseline samples. Key: t[1], principal component (PC) 1; t[2], principle component 2; R2X[1], PC1 variation within variables modeled; R2X[2], PC2 variation within variables modeled; MCT-KD, medium chain triglyceride ketogenic diet.

Canine feces analyzed by lipid profiling UPLC-MS in ESI positive mode resulted in the detection of 278 metabolic features, of which 155 were included in further data analysis after the application of quality filtering protocols. PCA multivariate modeling highlighted subtle clustering of samples based on diet phases (PCA scores plot, Figure 6). Further univariate analysis revealed 5 metabolic features which were significantly higher in abundance during the placebo diet when compared to the MCT-KD phase and 17 metabolic features which were significantly higher in abundance during both the MCT-KD and placebo diet phases when compared to the normal diet phase (Supplementary Table 6). Unfortunately, further metabolite feature identification by LC-MSMS fragmentation profiling were inconclusive. Although metabolite features were not confidently identified, the MSMS fragmentation patterns along with the large metabolite feature masses indicates that the 5 metabolite features significantly higher in abundance during the placebo diet belong to the LCT class of metabolites.

## Discussion

The primary aim of this study was to evaluate changes to the fecal microbiome and lipidome of dogs recruited on the MCT-KD trial (16). Intuitively, consumption of different diets are associated with corresponding differences in gut microbiome as shown in a number of biological models including humans, companion animals and research animals (21, 27, 38–40). As such, differentiating the changes in microbiome that are directly associated with the intended effect of diet, such as anti-seizure effect, to general diet related changes presents as one of the challenging aspects of diet-microbiome related research. Furthermore, interpretation of diet-microbiome related published literature must involve diligent consideration where significant changes to the diet itself inherently presents as a confounding factor to subsequent significant findings. The MCT-KD trial compares the MCT-KD to an isocaloric placebo diet, where the proportions of fats, proteins and carbohydrates remain identical with the acceptation that the MCT-KD contains 5.5% MCTs and the placebo contains lard in replacement (16). The minimal difference in MCT-KD and placebo diet components allows for evaluation of the MCT effects on gut microbiome while minimizing the confounding factor of macronutrient perturbation effects on the gut microbiome. Furthermore, any aforementioned effects resulting from macronutrient changes can be evaluated when making comparisons to baseline profiles prior to commencement of the MCT-KD trial.

Studies investigating the relationship between gut microbiome and disease states in dogs, such as inflammatory bowel disease, obesity and lymphoma, all show that decreases in alpha diversities within bacterial communities correlate with unfavorable disease outcomes when compared to healthy controls (41–44). Xie et al. showed that the Shannon alpha diversity index was higher in healthy infants when compared to infants with refractory epilepsy and that diversity was increased after a KD treatment period (45). Another human study investigating the relationship between KDs, epilepsy and gut microbiome showed that consumption of a KD when compared to baseline resulted in a reduction in alpha diversity, although this was reported as a trend which was not statistically significant (46). In one mice study, it was shown that consumption of a KD significantly reduced alpha diversity when compared to a control diet (27). As discussed, comparing changes in the microbiome between different studies investigating the effects of diets requires careful consideration of the diets being investigated. In the aforementioned studies, the fat:non-fat ratios in the KD ranged from 4:1 in the human studies to 6:1 in the mice study, therefore representing a macronutrient dietary shift when compared to baseline (27, 45, 46). In this study, species richness (α-diversity) of bacterial communities showed a statistically significant increase during the MCT-KD when compared to the baseline, but no effect was observed on placebo diets. The results presented in this study better represents the micronutrient effects, as the test diets were formulated to have similar dietary compositions, and their composition was also similar to that of commercially available dry kibble diets consumed at baseline. Up to date, studies reporting the effects of KDs have shown both increases and decreases in alpha diversity within bacterial communities. More studies are required to elucidate and characterize the effects of different KDs on microbial diversities.

Using a mice model of refractory epilepsy Olson et al. showed that consumption of a KD not only elevated the seizure threshold but also selectively increased the relative abundance of *Akkermansia muciniphila* and *Parabacteroides* (27). Furthermore, the anti-seizure protective effect of the KD-associated *A. muciniphila* and *Parabacteroides* was shown to be transferable to mice fed a non-KD (27). *A. municiphila* was not detected in this study and although *Parabacteroides* was absent in all dogs at baseline, it was later detected in one dog both during the consumption of the MCT-KD and placebo diet, and another dog only during consumption of the MCT-KD, a trend that did not reach significance (data not shown, *p* = 0.333). It has previously been shown that although microbial communities vary widely even among healthy individuals, the metagenomic carriage of metabolic pathways remain stable (47). An unnamed *Bacteroidaceae* species within genus *5-7N15* was identified by LEFSe in this study as a potential biomarker associated with consumption of the MCT-KD. The same genus was also shown to be higher in abundance during the MCT-KD via univariate tests, however lost statistical significance upon multiple comparison correction. *5-7N15*, a genus from the *Bacteroidaceae* family (phylum Bacteroidetes), has previously been described in humans, sheep, cows and dogs (48–51). In humans *57N15* was found to positively correlate with genus *Akkermansia*, highlighting the potential that they might occupy a similar niche (51). Differences in the specific bacterial taxa detected may represent the biological and metabolic differences between canines and mice, especially when considering the likely differences in lipid metabolism between the two. Furthermore, the potential anti-seizure pathways affected by the specific bacterial taxa highlighted by Olson et al. may be represented by other bacterial taxa in canines (27).

In addition to reducing seizure frequency, the MCT-KD utilized in this study was previously reported to reduce the behavioral factors “chasing” and “stranger directed fear” (16, 28). Recently, it was shown that lineages of the *Bacteroidacea* family, including an unidentified species within genus *5-7N15*, were increased in non-aggressive dogs (50). Other species within this family, such as *Bacteroides fragilis*, have also been shown to modulate mammalian behavior (52). Interestingly, although not statistically significant, there was a trend showing a reduction in the behavioral factors “stranger-directed aggression” and “owner-directed aggression” when dogs with IE were fed the MCT-KD utilized in this study (28). Increases of the unnamed *Bacteroidaceae* species within genus *5-7N15* during the MCT-KD period highlighted in this study may contribute to behavior modulating effects, such as reductions in aggression related behaviors. Besides the *Bacteroidaceae* family, Kirchoff et al. also showed that taxa within the class *Fusobacteria* and family *Fusobacteriaceae* to be more abundant in non-aggressive dogs (50). Interestingly, the results presented in this study also found an increased abundance of one unclassified genus within *Fusobacteriaceae* during the MCT-KD compared to baseline, with a similar trend in the placebo group. Taxa highlighted within the *Bacteroidaceae* and *Fusobacteriaceae* family may possess behavioral modulating effects.

On the other hand, higher levels of *Fusobacterium*, a large genus group within the *Fusobacteriaceae* family, has also been detected in other carnivore species (53–55) and dogs fed high protein diets (38, 40, 56). The *Fusobacteriaceae* family includes *Fusobacterium varium*, a bacterial species known to produce butyrate from protein sources, indicating that *Fusobacteriaceae* may be associated with protein digestion (57). Both the MCT-KD and placebo diet utilized in this study contained relatively high protein levels, and therefore the slight increase in *Fusobacteriaceae* observed in our data may be attributed to an increase in microbial communities linked to protein digestion.

An unclassified genus within the *Erysipelotrichaceae* family, instead, increased with the placebo diet and showed a trend toward increasing within MCT-KD. Given the increase in abundance in both diets when compared to baseline, it is likely that this unclassified genus responded to changes in macronutrient composition rather than MCT.

Similar results were observed with other bacterial taxa. The abundance of both *Megamonas* sp. and *Blautia* sp. was reduced during both MCT-KD and placebo diet. Genera *Megamonas* and *Blautia* are known short-chain fatty acid producers, fermenting carbohydrates into propionate (58). Unfortunately, the baseline diets in this study were not standardized, and the macronutrient composition was not the same for all dogs. Therefore, we cannot determine if the decreased abundance of these two species is due to a decrease in carbohydrate availability, or due to other factors. Genera *Megamonas* and *Blautia* are considered beneficial taxa, and their decrease has been observed in dogs with chronic enteropathies (59–61).

Although there were significant differences between some metabolite features detected by LC-MS, exact identification was not pursued because of the additional costs associated with conducting analyses for untargeted metabolomics. Furthermore, the data generated in this study originate from samples which were diluted during sample preparation protocols, explaining the low abundance of metabolite features detected by UPLC-MS. Despite this limitation some metabolite features were shown to be significantly different between diet groups. Five metabolite features, tentatively identified as long chain triglycerides, were significantly higher after consumption of the placebo diet, which likely reflects the difference in fatty acid profile with the extra 5.5% lard present in the placebo diet. Furthermore, the 17 metabolic features which were significantly higher in abundance during both the MCT-KD and placebo diet phases when compared to the baseline diet phase likely represents metabolic constituents found within the test diets. Since we were unable to confidently identify the metabolic features that were significant, it is difficult to elucidate or infer further hypotheses. The recruitment criterion adopted by the diet trial study resulted in a pragmatic cohort population including different canine breeds. Although a crossover study design was adopted to capture perturbations in gut microbiota, authors acknowledge the potential that there may be variability in global gut microbiota reactions to the diets based on breed, which would not be captured in this study. More work is needed to interrogate the influence of ketogenic diets on the gut metabolome.

MCT-KD, which is a promising treatment for drug-resistant idiopathic epilepsy in dogs, does not significantly affect the overall gut microbial communities. MCT-KD did, however, increase bacterial richness, a finding commonly associated with successful treatment in other diseases. We were unable to identify *Akkermansia* in any of the fecal samples included in the study, which is a genus associated with successful KD treatment in humans and mice. However, bacterial genus *X57N15*, which was determined to positively correlate with *Akkermansia* in humans, was found to be significantly increased in dogs receiving MCT-KD, suggesting they occupy a similar niche. *Parabacteroides*, another genus associated with successful KD treatment in mice, was found only in two dogs, one during placebo and MCT-KD, and another only during MCT-KD. Interestingly, both dogs were negative for *Parabacteroides* before the trial. Furthermore, we previously reported behavioral changing effects that were associated with the MCT-KD, and based on these data may manifest due to changes to the microbiome. More studies are required to elucidate further the influences of MCTs on the microbiome and metabolome and the potential anti-seizure and behavioral modulating effects of specific microbial communities.

## Data Availability Statement

Raw DNA sequences were uploaded to NCBI Sequence Read Archive under the accession number SRP162687.

## Ethics Statement

The studies involving animals were reviewed and approved by the Royal Veterinary College Ethics and Welfare Group (URN 2011 1132).

## Author Contributions

RP compiled the 16S rRNA profiling data, led the statistical analysis of the data, interpreted the results, and wrote the manuscript. TL compiled the clinical data, acquired the UPLC-MS and 16S rRNA profiling data, led the statistical analysis of the UPLC-MS data, interpreted the results, and wrote the manuscript. EW contributed to acquisition of UPLC-MS data, consulted on statistical analysis, interpretation of data, and contributed to revisions of the manuscript. YP, BZ, JL, JMS, and QL contributed to revisions of manuscript. HV and JSS consulted on interpretation of results and contributed to revisions of the manuscript. All authors contributed to the article and approved the submitted version.

## Conflict of Interest

YP, BZ, and QL were employed by the company Nestlé Purina Research. YP and BZ are inventors of US9789079B2. The remaining authors possesses any rights of the patent. The commercial sponsor has not been involved in case recruitment, data handling, data analysis, and data storage. Furthermore, the commercial sponsor could not prevent the manuscript from being submitted for publication.
