# Supplementary material for: The Effects of a Ketogenic Medium-Chain Triglyceride Diet on the Feces in Dogs With Idiopathic Epilepsy
Source: Front Vet Sci. 2020 Dec 22;7:541547. doi: 10.3389/fvets.2020.541547 (PMC7783044; doi:10.3389/fvets.2020.541547)
Supplement: Supplementary file 1 [file Table_1.DOCX]

| **Dog ID** | **Dog breed** | **Age (years.months)** | **Weight (kg)** | **Sex (M/F)** | **Neuter status (Y/N)** |  |
| --- | --- | --- | --- | --- | --- | --- |
| **02** | English Springer Spaniel | 5.5 | 21.60 | FEMALE | YES |  |
| **23** | Saint Bernard | 3.8 | 62.40 | FEMALE | NO |  |
| **12** | Welsh Springer Spaniel | 4.3 | 25.15 | MALE | YES |  |
| **13** | Beagle | 6.8 | 14.95 | MALE | YES |  |
| **14** | American Bulldog | 4.3 | 33.80 | MALE | YES |  |
| **19** | Border Collie | 4.3 | 17.30 | MALE | YES |  |
| **21** | Cavalier King Charles | 4.7 | 10.20 | MALE | YES |  |
| **22** | Mastiff | 5.7 | 63.70 | MALE | YES |  |
| **25** | German Shepherd | 3.8 | 36.75 | MALE | NO |  |
| **33** | Slovakian Rough Haired Pointer | 5.5 | 36.95 | MALE | YES |  |
| **15** | Boxer | 6.2 | 30.15 | FEMALE | NO | Mean age = 4.34 (SD 2.25) years  Mean weight = 27.52 (SD 10.51)kg |
| **17** | English Bull Terrier | 2.4 | 29.90 | FEMALE | YES |  |
| **20** | Cross breed | 2.2 | 25.90 | FEMALE | YES |  |
| **28** | Ihasn Aspo | 3.0 | 9.45 | FEMALE | YES |  |
| **05** | Golden retriever | 3.2 | 38.90 | MALE | NO |  |
| **06** | Cross breed | 2.2 | 26.50 | MALE | YES |  |
| **09** | Rhodesian Ridgeback | 4.3 | 46.30 | MALE | NO |  |
| **16** | Border Collie | 4.6 | 26.70 | MALE | NO |  |
| **27** | Cross breed | 9.7 | 13.50 | MALE | YES |  |
| **29** | Siberian Huskey | 4.2 | 34.10 | MALE | YES |  |
| **30** | Beagle | 5.9 | 21.30 | MALE | NO |  |

**Supplementary Table 1.** Breed, age, weight, sex and neuter status of all dogs included in the MCT-KD diet trial study. Highlighted in grey are all dogs included in this study, where all other dogs were excluded due to antibiotic administration during the MCT-KD diet trial. Mean age and weight of dogs included in this study are summarized standard deviation.
