# Supplementary material for: The Effects of a Ketogenic Medium-Chain Triglyceride Diet on the Feces in Dogs With Idiopathic Epilepsy
Source: Front Vet Sci. 2020 Dec 22;7:541547. doi: 10.3389/fvets.2020.541547 (PMC7783044; doi:10.3389/fvets.2020.541547)
Supplement: Supplementary file 2 [file Table_2.DOCX]

| **Lipid profiling –UPLC-MS** |
| --- |
| Fecal samples (30mg) were combined with 900μl pre-chilled (-20ºC) methanol/HPLC-grade water (1:1) in homogenizing bead beater tubes containing ~50mg of 1mm zirconia silica beads (BioSpec Products Inc, USA). Samples were homogenized using a Precellys bead beater for 2 cycles of 40 second at 6,500Hz speed per cycle. Samples were cooled on ice between homogenizing cycles. Homogenized samples were then centrifuged at 10,000g for 10 minutes at 4ºC. 600μl of the supernatant was removed for analysis which is not included in this study. 1ml of pre-chilled dichloromethane/methanol (3:1) was combined with the contents remaining in the bead beater tubes and further homogenized for 2 cycles using the same parameters described. Samples were centrifuged at 10,000g for 10 minutes at 4ºC. Supernatants were collected into 2ml MS glass vials and dried in a fume cupboard at room temperature (~20ºC). Dried extracts were re-suspended in 200μl of methanol/water (1:1), vortex mixed and sonicated at room temperature. 100μl of each sample was transferred into glass inserts with polystring (Waters Ltd., UK) and placed inside 2ml MS glass vials ready for UPLC-MS analysis. |
| **16S rRNA gene sequencing** |
| A fecal aliquot was used for DNA extraction using a Biostatic Bacteremia DNA Isolation Kit (MoBio Laboratories, USA) following the manufacturer's instructions. Extracted DNA was quantified using PicoGreen® dsDNA Assay Kit (Life Technologies Corporation) and read on a BioTek FLX800 microplate fluorescent reader (Biotek Instruments Inc.). Furthermore, quality of extracted DNA was checked using E-Gel® 96 Agarose Gels (0.1%), E-Gel® 96 High range DNA Maker and run on a Mother E-Base device (Life Technologies, Corp.). Unique sequence barcodes to identify individual samples, reverse primers and forward primers were added, followed by polymerase chain reaction (PCR) protocols to amplify extracted DNA. (Fusion Primers (forward and reverse) purchased from Integrated DNA technologies (IDT)). The PCR run cycles were as follows; initial denaturing of double stranded DNA, 1 cycle 94^o^C for 2 minutes; DNA denaturing, annealing and elongation, 25 cycles of 94^o^C for 30 seconds, 49^o^C for 30 seconds and 72^o^C for 1 minute; final DNA elongation, 1 cycle of 72^o^C for 7 minutes. PCR product was quantified using PicoGreen® dsDNA Assay Kit (Life Technologies Corporation). Removal of primers and irrelevant small fragments of DNA were carried out using Purelink® Pro 96 Purification Kit (Life technologies Corporation). Purified PCR product was quantified using PicoGreen® dsDNA Assay Kit (Life Technologies Corporation) and read on a BioTek FLX800 microplate fluorescent reader (Biotek Instruments Inc.). Quality of extracted DNA was checked using E-Gel® 96 Agarose Gels (0.2%), E-Gel® 96 High range DNA Maker and run on a Mother E-Base device (Life Technologies, Corp.). DNA sequences were pooled and normalized. Quality of pooled DNA was checked using High Sensitivity DNA Analysis Kits (Agilent Technologies) and read on a Bioanalyzer 2100 (Agilent Technologies). Emulsion PCR (emPCR; Roche Diagnostics) was performed, and after the number of beads was normalized, DNA sequencing was ran using a 454 GS-FLX+ Genome Sequencer (Roche Diagnostics). DNA sequence data was generated and exported in the FASTA file format containing multiplexed DNA sequence information. |
|  |

**Supplementary Table 2.** Canine fecal sample collection and preparation protocol.
