# Supplementary material for: The Effects of a Ketogenic Medium-Chain Triglyceride Diet on the Feces in Dogs With Idiopathic Epilepsy
Source: Front Vet Sci. 2020 Dec 22;7:541547. doi: 10.3389/fvets.2020.541547 (PMC7783044; doi:10.3389/fvets.2020.541547)
Supplement: Supplementary file 3 [file Table_3.DOCX]

| **Ultra Performance Liquid Chromatography** | | | | | |
| --- | --- | --- | --- | --- | --- |
| Chromatography column: | | Acquity UPLC C_18_ CSH (Charged Surface Hybrid) column (2.1mm x 100mm, 1.7μm particles; Waters Corporation, USA). Column maintained at 55^o^C throughout analytical protocol. | | | |
| Mobile phase A: (ACN:H_2_O (v/v) (60:40), 0.1% (v/v) FA, 10mM ammonium formate) | | | | | |
| Mobile phase B: (IPA:ACN (90:10), 0.1% (v/v) FA, 10mM ammonium formate) | | | | | |
| Stage | Time (mins) | | Mobile Phase A / (%) | Mobile Phase B / (%) | Flow rate (mL/min) |
| Initial | 0.00 | | 60 | 40 | 0.5 |
| 1 | 2.00 | | 57 | 43 | 0.5 |
| 2 | 2.10 | | 50 | 50 | 0.5 |
| 3 | 12.00 | | 46 | 54 | 0.5 |
| 4 | 12.10 | | 30 | 70 | 0.5 |
| 5 | 18.00 | | 1 | 99 | 0.5 |
| 6 | 18.10 | | 60 | 40 | 0.5 |
| 7 | 20.00 | | 60 | 40 | 0.5 |
| **Mass Spectrometer Parameters in UPLC-MS Experiments ( Q-TOF Premier)** | | | | | |
| ESI (+) Capillary Voltage [kV] | | | | 3.0 | |
| ESI (-) Capillary Voltage [kV] | | | | 2.5 | |
| ESI (+) Sample Cone Voltage [V] | | | | 30 | |
| ESI (-) Sample Cone Voltage [V] | | | | 25 | |
| Source Temperature [^o^C] | | | | 120 | |
| Desolvation Temperature [ ^o^C] | | | | 400 | |
| Cone Gas Flow [L/hour] | | | | 25 | |
| Desolvation gas flow [L/hour] | | | | 800 | |
| Scan Range [Da] | | | | 50-1200 | |
| Scan Duration [sec] | | | | 0.3 | |
| **Data Processing Parameters (XCMS)** | | | | | |
| library(xcms) | | | | | |
| <- xcmsSet(method="centWave", peakwidth=c(4,16), ppm=70, snthresh=10, integrate=2, prefilter=c(5,300), noise=100, mzCenterFun="wMean") | | | | | |
| <- retcor(pp, method="obiwarp", plottype = "d") | | | | | |
| <- group(cpp, method="density", bw=5, mzwid=0.03, minfrac=0.5, minsamp=5) | | | | | |
| <- fillPeaks(gcpp) | | | | | |
| A coefficient of variation of ≥30% within QCs for all extracted metabolite features formed the major criteria for removal of metabolite features that were considered unreliable. Furthermore, metabolite features that were not present in (QCn-1) QC samples were also considered unreliable and removed from further data analysis. | | | | | |

**Supplementary Table 3**. Parameters utilized in metabolic profiling analysis. Gradient program for chromatography of reversed phase lipid profiling (LP-)UPLC-MS. Sample injection volume was set to 5μl per injection. All reagents/solvents were LC-MS grade. Using the LockSpray interface (Waters Corporation) leucine encephaline (m/z of 556.277 (ESI+), 554.262 (ESI-)), (200pg/μl in 50:50 (v/v) CH_3_CN:H_2_O, 0.1% (v/v) FA) was infused at 20μl/min as the lock mass reference to optimize instrument mass accuracy. Mass spectrometer chromatograms and spectra were visualized using MassLynx software (version 4.1, Waters Corporation). Raw data files generated by UPLC-MS experiments were converted to NetCDF for further processing using Databridge software implemented in MassLynx software. Further spectral processing was carried out using R programming language (open-source software) and XCMS software package. Key; Stage, distinctive stage of gradient change initiation; Time, time during analytical run; LP, lipid profiling; H_2_O, water (Fisher Scientific); FA, formic acid (Sigma, USA); ACN, Acetonitrile (Honeywell); IPA, Isopropanol (Honeywell); kV, kilovolts; V, volts; ^o^C, degree Celsius; L, liters; Da, Daltons; sec, seconds.
