# Supplementary material for: The Effects of a Ketogenic Medium-Chain Triglyceride Diet on the Feces in Dogs With Idiopathic Epilepsy
Source: Front Vet Sci. 2020 Dec 22;7:541547. doi: 10.3389/fvets.2020.541547 (PMC7783044; doi:10.3389/fvets.2020.541547)
Supplement: Supplementary file 4 [file Table_4.DOCX]

| **Seizure frequency per month (p=0.148)** | | | |
| --- | --- | --- | --- |
| **DOG ID** | **Placebo** | **MCT-KD** | **Change (%)** |
| **15** | 2.02 | 0.00 | -100 |
| **29** | 0.65 | 0.00 | -100 |
| **28** | 2.28 | 0.66 | -71 |
| **17** | 22.92 | 9.89 | -57 |
| **06** | 11.61 | 5.67 | -51 |
| **20** | 2.67 | 1.33 | -50 |
| **09** | 1.89 | 1.00 | -47 |
| **27** | 5.39 | 3.30 | -39 |
| **16** | 2.02 | 2.28 | 13 |
| **30** | 3.37 | 5.00 | 48 |
| **05** | 4.43 | 8.00 | 81 |
| **Seizure day frequency per month (p=0.054)** | | | |
| **DOG ID** | **Placebo** | **MCT-KD** | **Change (%)** |
| **15** | 1.69 | 0.00 | -100 |
| **29** | 0.65 | 0.00 | -100 |
| **28** | 1.30 | 0.33 | -75 |
| **09** | 1.58 | 0.67 | -58 |
| **17** | 13.82 | 7.58 | -45 |
| **27** | 4.04 | 2.31 | -43 |
| **30** | 2.70 | 2.00 | -26 |
| **20** | 1.67 | 1.33 | -20 |
| **16** | 1.01 | 0.98 | -3 |
| **06** | 1.61 | 2.00 | 24 |
| **05** | 2.39 | 4.00 | 68 |

**Supplementary Table 4**. Overview of seizure frequency per month and seizure day frequency per month in each dog during the placebo diet and MCT-KD respectively (n=11). Overall response is represented by the percentage change between outcome variables respectively. Wilcoxon matched paired t-tests were used to make comparisons between diet groups. Statistical significances between diets are presented as p-values and highlighted in blue
