# Supplementary material for: The Effects of a Ketogenic Medium-Chain Triglyceride Diet on the Feces in Dogs With Idiopathic Epilepsy
Source: Front Vet Sci. 2020 Dec 22;7:541547. doi: 10.3389/fvets.2020.541547 (PMC7783044; doi:10.3389/fvets.2020.541547)
Supplement: Supplementary file 6 [file Table_6.docx]

**Supplementary Table 6.** Metabolites shown to be significantly different in abundance between placebo diet and MCT-KD phases in fecal samples. Metabolites were detected in ESI+ and ESI- modes using LP-UPLC-MS and RP-UPLC-MS. Significant metabolites were determined using paired student t-test with FDR p-value correction, (p<0.05) and identified in UPLC-MS/MS experiments respectively. Key: M, MCT-KD phase; P, Placebo diet phase; Met, metabolite; Δppm, deviation between measured mass and theoretical mass in ppm calculated; CV(%), coefficient of variation calculated based on total number of pooled QC samples acquired during experimental run; FDR, false discover rate p-value correction; m/z, mass-to-charge ratio; ESI, electro spray ionisation; PC, phosphocholine; FA, Formate; RP, reversed phase; LP, Lipid profiling.

|  | **MET Feature Mass** | **MET Feature RT** | **MCT-KD vs PLACEBO** | | | |
| --- | --- | --- | --- | --- | --- | --- |
| **MET ID** |  |  | **p** | | **p(FDR)** | |
| M879T912 | 878.7316 | 15.20 | <0.001 | | 0.023 | |
| M878T912 | 877.7300 | 15.20 | <0.001 | | 0.023 | |
| M904T913 | 903.7480 | 15.22 | <0.001 | | 0.023 | |
| M880T928 | 879.7454 | 15.47 | <0.001 | | 0.023 | |
| M906T929 | 905.7631 | 15.49 | <0.001 | | 0.023 | |
|  | **MET Feature Mass** | **MET Feature RT** | **MCT-KD vs NORM** | | **PLACEBO vs NORM** | |
| **MET ID** |  |  | **p** | **p(FDR)** | **p** | **p(FDR)** |
| M569T33 | 569.3163 | 32.70 | <0.001 | 0.002 | <0.001 | 0.020 |
| M525T33 | 525.2894 | 32.70 | <0.001 | 0.004 | 0.001 | 0.020 |
| M586T33 | 586.2953 | 33.11 | <0.001 | 0.001 | 0.002 | 0.020 |
| M717T33 | 717.3690 | 33.11 | <0.001 | 0.004 | 0.003 | 0.020 |
| M630T33 | 630.3204 | 33.13 | <0.001 | 0.002 | 0.007 | 0.038 |
| M629T33 | 629.3141 | 33.13 | <0.001 | 0.003 | 0.003 | 0.022 |
| M673T33 | 673.3422 | 33.13 | <0.001 | 0.003 | 0.007 | 0.038 |
| M542T33 | 542.2671 | 33.15 | <0.001 | 0.001 | 0.002 | 0.020 |
| M541T33 | 541.2632 | 33.16 | <0.001 | 0.002 | 0.004 | 0.023 |
| M585T33 | 585.2894 | 33.16 | <0.001 | 0.001 | 0.003 | 0.020 |
| M497T33 | 497.2363 | 33.17 | <0.001 | 0.003 | 0.002 | 0.020 |
| M608T33 | 608.3877 | 33.17 | <0.001 | 0.001 | 0.002 | 0.020 |
| M498T34 | 498.2428 | 33.56 | <0.001 | 0.002 | 0.003 | 0.020 |
| M453T34 | 453.2068 | 33.57 | <0.001 | 0.003 | 0.001 | 0.020 |
| M564T34 | 564.3611 | 33.58 | <0.001 | 0.001 | 0.002 | 0.020 |
| M483T34 | 483.1919 | 34.10 | 0.008 | 0.042 | <0.001 | 0.020 |
| M485T95 | 485.3605 | 94.80 | <0.001 | 0.002 | 0.002 | 0.020 |
